# Cost-effectiveness of community-based interventions for type 2 diabetes management in Indonesia: a time-horizon sensitivity analysis

**DOI:** 10.1186/s12962-026-00812-2

**Published:** 2026-08-05

**Authors:** Aalbrecht A. Irawan, Nursanti Anggriani, Yudhie Andriyana, Rizky Abdulah

**Affiliations:** 1 Doctoral Program in Mathematics, Faculty of Mathematics and Natural Science, Universitas Padjadjaran, Jalan Ir Soekarno KM 21, West Java 45363 Sumedang, Indonesia; 2 Department of Mathematics, Faculty of Mathematics and Natural Science, Universitas Padjadjaran, Jalan Ir Soekarno KM 21, West Java 45363 Sumedang, Indonesia; 3 Department of Statistics, Faculty of Mathematics and Natural Science, Universitas Padjadjaran, Jalan Ir Soekarno KM 21, West Java 45363 Sumedang, Indonesia; 4 Department of Pharmacological and Clinical Pharmacy, Faculty of Pharmacy, Universitas Padjadjaran, Jalan Ir Soekarno KM 21, West Java 45363 Sumedang, Indonesia

**Keywords:** Health policy, Resource allocation, Mathematic modeling, Lifestyle modification, Diabetes management strategies

## Abstract

**Background:**

Type 2 Diabetes Mellitus (T2DM) imposes a severe economic burden on Indonesia’s Social Health Insurance Administration Body (BPJS Kesehatan), driven predominantly by expensive late-stage complications. While the Prolanis program offers community-based prevention, a lack of long-term economic forecasting hinders optimal resource allocation among its core interventions. To address this gap, this study aims to evaluate community-based intervention strategies to determine which approach is the most cost-effective for long-term T2DM management in Indonesia.

**Methods:**

We developed a deterministic linear compartmental state-transition model to forecast the 25-year epidemiological and economic impacts of six intervention strategies derived from three Prolanis pillars: health education, physical activity, and lifestyle modification. The model integrated an economic resource allocation framework, Indonesian epidemiology data, and a 3.8-fold complication cost penalty multiplier for complication states. Cost-effectiveness was evaluated from a healthcare payer perspective using the Incremental Cost-Effectiveness Ratio (ICER) and Time-Horizon Sensitivity Analysis.

**Results:**

Over 25 years, the Lifestyle Modification intervention emerged as the dominant strategy compared to baseline care. It generated the highest increase in the managed T2DM population while incurring the lowest total systemic cost. ICER analyses demonstrated that Lifestyle Modification strictly dominated individual and combined promotive activities, which were subject to severe budgetary restrictions due to their high delivery cost-weights. Furthermore, temporal analysis confirmed that Lifestyle Modification achieves systemic net savings rapidly by Year 5. By Year 25, it resulted in projected cumulative net savings of 19.22 trillion IDR under the 3% base-case discount rate, with sensitivity analyses confirming robust dominance across 0% (27.61 trillion IDR savings) and 5% (15.48 trillion IDR savings) discount rates.

**Conclusion:**

Lifestyle Modification is the most economically dominant community-based intervention for managing T2DM in Indonesia. To avert the financial penalties of downstream complications, policymakers should strategically rebalance the Prolanis capitation budget to prioritize proactive, monitoring-driven lifestyle interventions.

## Background

Type 2 diabetes mellitus (T2DM) poses an increasingly unsustainable economic and clinical burden globally, with costs driven predominantly by the high prevalence and progression of late-stage microvascular and macrovascular complications [1]. In Indonesia, this global trend translates into severe pressure on the national healthcare system. Recent Indonesian macroeconomic evaluations and cohort-based studies by Hidayat et al. (2022) and Ramadani et al. (2024) reveal that complications account for most diabetes-related expenditures in Indonesia, with 74% of total T2DM spending incurred by patients with complications, and annual per-patient costs more than doubling when complications are present [2, 3]. The scale of morbidity is similarly substantial: 84.4% of Indonesians with T2DM have at least one complication, while 28.7% experience both microvascular and macrovascular complications, reflecting long-standing poor glycemic control and delayed detection [4]. Cardiovascular disease is the most common and costly complication nationally, affecting nearly one-quarter of patients and increasing healthcare utilization up to 9.9-fold [2]. Neuropathy further contributes to disability and healthcare demand, with peripheral neuropathy increasing hospital visits by 46% and substantially raising direct medical costs [5]. Diabetic nephropathy remains a leading cause of chronic kidney disease and end-stage renal disease, generating high inpatient expenditures and requiring resource-intensive renal replacement therapy [6]. These findings underscore the urgent need for strengthened early detection and comprehensive risk-factor management to mitigate the escalating burden of T2DM on Indonesia’s healthcare system.

To mitigate this burden, the Social Health Insurance Administration Body (BPJS Kesehatan) implemented the Chronic Disease Management Program (Prolanis). Operating at primary healthcare facilities, Prolanis is designed to proactively manage chronic conditions to prevent severe complications through structured community-based primary care services [7]. The program rests on several core community-based pillars, most notably health education/consultation, group physical activity, and routine health status monitoring [8]. 

While Prolanis has proven effective in short-term clinical evaluations, comprehensive long-term economic forecasting of the program remains limited [9]. Current economic evaluations of community-based health interventions, particularly in low- and middle-income countries (LMICs), are frequently constrained by a reliance on small-scale clinical trials and specific cohorts [10–13]. While randomized controlled trials provide precise data, their results are often difficult to generalize to broader national populations or diverse geographic settings, which limits their utility for large-scale policy decisions [10]. Furthermore, some existing modeling frameworks allow reversible transitions or simplified disease pathways that may not fully reflect the chronic and irreversible accumulation of late-stage complications [14–16]. Such structural assumptions can lead to an overestimation of long-term cost-effectiveness when delayed complication costs and progressive disease trajectories are insufficiently captured [12, 17]. 

Beyond these modeling limitations, economic evaluations of community-based health interventions often lack direct relevance to the specific needs of LMIC policymakers. Many studies fail to define a clear decision-making context or neglect essential equity impacts and broader social outcomes [11, 12, 18]. These gaps are compounded by inconsistent guidelines, a lack of robust sensitivity analyses, and significant regional underrepresentation [19, 20]. To provide more actionable evidence, more research should move beyond context-bound estimates by integrating more realistic disease trajectory modeling and aligning evaluation frameworks with local policy priorities [12, 19].

To address this critical gap in the health economics literature, this study aims to determine which community-based intervention strategy under Indonesia’s Prolanis program is the most cost-effective for long-term T2DM management. To achieve this goal, we evaluate six mutually exclusive community-based health interventions derived from the core Prolanis pillars: health education, physical activity, and lifestyle modification. By employing a deterministic linear state-transition compartmental model, we project the epidemiological and economic dynamics of T2DM over a 25-year horizon. To accurately simulate fiscal boundaries, this study utilizes a resource allocation framework, which is defined here as the mathematical integration of intervention cost-weights and optimal control variables used to dynamically constrain budget intensity.

## Methods

### Model structure and compartmental dynamics

This study used a deterministic linear compartmental model to project the epidemiological and economic dynamics of T2DM. While probabilistic Markov state-transition models are a frequently used methodological approach for projecting long-term diabetes complication costs, our study adapts this concept into a continuous deterministic compartmental framework to model dynamic optimal control [21]. 

The epidemiological compartmental structure and core transition parameters were adapted from our previously developed non-reversible T2DM optimal-control model, which established the mathematical well-posedness, positivity, boundedness, and stability of the system [22]. The present study extends that model by adding a payer-perspective health-economic evaluation, discounted cumulative costs and outcomes, ICER analysis, and time-horizon sensitivity analysis.

**Fig. 1:**
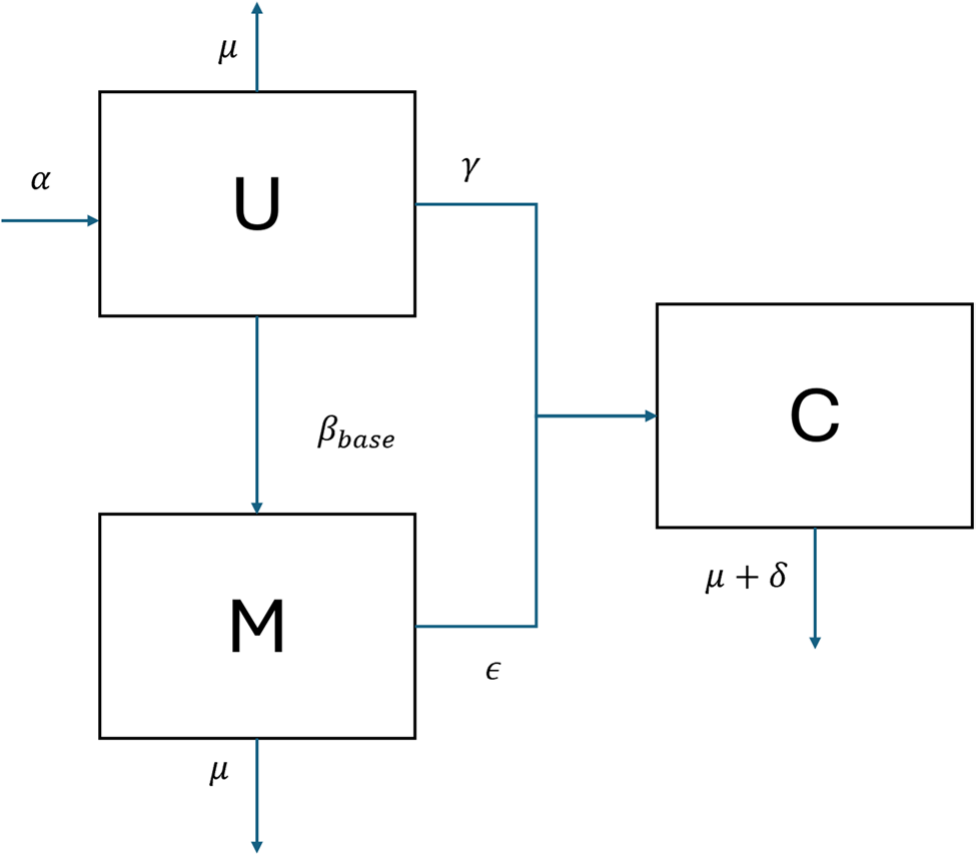
Model structure and state transitions

The population is divided into three mutually exclusive health states: unmanaged T2DM (*\:U*), managed T2DM (*\:M*), and T2DM with complications (*\:C*). The model structure and state transitions are illustrated in Fig. 1, in accordance with CHEERS reporting recommendations for model-based economic evaluation. To accurately reflect the chronic clinical trajectory of the disease and prevent the overestimation of cost-effectiveness, the progression from the unmanaged and managed states to the complication state is modeled as strictly irreversible. The baseline continuous-time transitions between these compartments prior to the optimization of active interventions, are governed by the following system of linear ordinary differential equations (ODEs):

$$\:\frac{dU}{dt}=\alpha\:-\left(\mu\:+\gamma\:+{\beta\:}_{base}\right)U,$$

$$\>{{dM} \over {dt}} = \beta {\>_{base}}U - \left( {\epsilon + \mu \>} \right)M,$$

$${{dC} \over {dt}} = \gamma U + \epsilon M - \left( {\delta + \mu } \right)C,$$

where $\:\alpha\:$ represents the incidence of new T2DM cases, $\:\mu\:$ is the natural mortality rate, and $\:\delta\:$ is the complication-specific mortality rate. The parameters $\:\gamma\:$ and *ε* denote the natural transition rates into complications from the unmanaged and managed populations, respectively. The parameter $\:{\beta\:}_{base}$ represents the natural transition rate to the managed state without amplified program intensity.

### Data sources and parameters

To forecast the long-term economic impact of community-based diabetes interventions, this study synthesized epidemiological and economic inputs from secondary Indonesian data sources, including national health-survey data, cohort studies, T2DM burden projections, and published economic evaluations. This parameterization incorporates the specific variables used for cost and effect inputs, model assumptions, and the discounting approach applied over the 25-year projection horizon (Table 1). Because this study used secondary data, unsourced operational details such as individual attendance, adherence behavior, implementation fidelity, and facility-level delivery frequency were not imputed. Individual-level adherence was not modeled as a dynamic behavioral feedback variable. Instead, the transition parameters and intervention modifiers represent population-level effects derived or calibrated from available secondary data and therefore may only indirectly reflect average implementation uptake.

**Table 1:** Secondary data sources and model parameters for 25-year forecasting

Parameter	Variable	Value	Unit	Source
Initial unmanaged T2DM population	$\:{U}_{0}$	12.4	Million persons	Calculated from [23,24] Parameterization adapted from [22]
Initial managed T2DM population	$\:{M}_{0}$	4.75	Million persons	[23] Parameterization adapted from [22]
Initial T2DM population with complications	$\:{C}_{0}$	6	Million persons	[23] Parameterization adapted from [22]
New Incidence of T2DM	$\:\alpha\:$	0.97	Million year^− 1^	[23] Parameterization adapted from [22]
Natural mortality	$\:\mu\:$	0.105648	year^− 1^	[23] arameterization adapted from [22]
Complication-related mortality	$\:\delta\:$	0.01168	year^− 1^	Parameterization adapted from [22]
Unmanaged progression to Complications	*γ*	0.42098	year^− 1^	Calculated from [24] Parameterization adapted from [22]
Managed progression to Complications	*\:ϵ*	0.042098	year^− 1^	Parameterization adapted from [22]
Health education transition rate	$\:{\beta\:}_{E}$	0.011307	year^− 1^	[23] Parameterization adapted from [22]
Physical activity transition rate	$\:{\beta\:}_{A}$	0.006228	year^− 1^	[23] Parameterization adapted from [22]
Lifestyle modification rate	$\:{\beta\:}_{L}$	0.012157	year^− 1^	[23] Parameterization adapted from [22]
Baseline care tariff	*\:-*	427	USD per patient-year	[25]
Complication penalty cost	*\:-*	1,607	USD per patient-year	[25]
Health education cost-weight	$\:{A}_{E}$	0.726289	Dimensionless	[8] Parameterization adapted from [22]
Physical activity cost-weight	$\:{A}_{A}$	0.451913	Dimensionless	[8] Parameterization adapted from [22]
Lifestyle modification cost-weight	$\:{A}_{L}$	0.005151	Dimensionless	[8] Parameterization adapted from [22]
Maximum implementation intensity	$\:{u}_{max}$	5	Dimensionless	Parameterization adapted from [22]
Objective function benefit weight	*\:B*	1	Dimensionless	Parameterization adapted from [22]
Discount rate	*\:-*	$\:3\%$	Annual	[26]

Note: Core epidemiological and optimal-control parameters were adapted from the previously developed deterministic T2DM model. The current study did not repeat the mathematical stability proofs. Instead, it extended the accepted model into a health-economic framework by incorporating direct medical costs, complication-cost penalties, discounting, and ICER-based decision rules

### Optimal control and community-based interventions

The transition of patients from the unmanaged (*\:U*) to the managed (*\:M*) compartment is driven by community-based interventions implemented under BPJS Kesehatan’s Prolanis program:

- Health education ($\:{\beta\:}_{E}$): Structured monthly medical consultations and health education sessions.
- Physical activity ($\:{\beta\:}_{A}$): Community-led Prolanis club activities and group exercises.
- Lifestyle modification ($\:{\beta\:}_{L}$): Monitoring-supported behavioral and risk-factor management based on routine assessment of clinical markers and health status monitoring. This intervention is considered low-cost in the model because it is embedded within routine primary-care workflows, avoiding the additional logistical, facility, and coordination expenses associated with organizing group physical activities.

Because the analysis used secondary data, intervention definitions were restricted to published Prolanis descriptions and their corresponding model representation. No additional assumptions were made regarding actual attendance, individual adherence, or implementation fidelity beyond the available secondary evidence.

To model the dynamic implementation intensity of the interventions, we introduced time-dependent control variables ($\:{u}_{i}$). These variables represent the dynamically optimized implementation intensity for each strategy ($\:i\in\:\left\{E,\:L,\:A\right\}$). The control variable acts as an additive scalar to the specific intervention’s efficacy, modifying the transition rate under an active strategy as follows:

$$\:{\beta\:}_{active}={\beta\:}_{base}+{\beta\:}_{i}{u}_{i}.$$

When an intervention strategy is activated, the system dynamics are updated to incorporate this optimal control effort:

$$\:\frac{dU}{dt}=\alpha\:-(\mu\:+\gamma\:+{\beta\:}_{active})U$$

$$\:\frac{dM}{dt}={\beta\:}_{active}U-(\epsilon +\mu\:)M$$

$$\:\frac{dC}{dt}=\gamma\:U+\epsilon M-\left(\delta\:+\mu\:\right)C$$

To determine the most efficient use of the Prolanis budget, we evaluated six mutually exclusive community-based intervention strategies alongside the baseline:

1. Physical activity only.
2. Health education only.
3. Lifestyle modification only.
4. Lifestyle modification + physical activity.
5. Lifestyle modification + health education.
6. Health education + physical activity.

To prevent the assumption of unlimited resources, the feasible intensity of each strategy was dynamically constrained by its economic cost-weight. To solve for the optimal feasible time-varying implementation intensity under these budget and cost-weight constraints, the model utilized Pontryagin’s Maximum Principle, employing the Forward-Backward Sweep Method over 2,500 iterations to generate the optimal trajectories for $\:{u}_{i}\left(t\right)$ prior to the economic evaluation [27]. 

### Economic evaluation and total system cost

Following the optimization, a post-hoc economic evaluation was conducted to determine the ICER. Costs were evaluated from a healthcare payer perspective, capturing direct medical treatment expenditure and primary-care delivery costs. The unit cost-weights for delivering health education ($\:{A}_{E}$), physical activity ($\:{A}_{A}$) and lifestyle modification ($\:{A}_{L}$) at the primary care level were derived from existing Indonesian macroeconomic evaluations of the Prolanis program [8]. Specifically, group-based activities such as health education ($\:{A}_{E}=0.726289$) and physical activity ($\:{A}_{A}=0.451913$) carry substantially higher delivery cost-weights because they required dedicated facility space, coordination logistics, and structured healthcare personnel hours [8]. Conversely, lifestyle modification ($\:{A}_{L}=0.005151$) carries a minimal cost-weight because it is integrated into routine primary care clinical consultations and health-status monitoring workflows, avoiding these additional group coordination overheads [8]. 

A critical component of this economic evaluation is the complication penalty cost assigned to the complication compartment (*\:C*). Recent systematic evaluations in Indonesia establish that the weighted average direct medical cost for a patient with complications is USD 1,607 annually, approximately 3.8 times higher than the USD 427 required for a patient without complications [25]. 

To determine the cost-effectiveness of the optimal trajectories, the Total Cost for each strategy was calculated by integrating the baseline care tariff (weighted at 1.0 for unmanaged/managed patients), the 3.8-fold complication penalty multiplier, and the quadratic program delivery costs ($\:{A}_{i}{u}_{i}^{2}$) over the 25-year projection period (*\:T=25*):

$$Total\>Cost = \int \> _0^T\left[ \matrix{ 1.0\left( {U + M} \right) + 3.8C \hfill \cr + {A_E}u_E^2 + {A_L}u_L^2 + {A_A}u_A^2 \hfill \cr} \right]dt.$$

To maintain numerical stability within the deterministic optimization framework, economic expenditures and cumulative cost outputs are reported in dimensionless model cost units, where 1.0 model cost units correspond to the IDR-trillion reporting scale derived from baseline tariff parameters, which is the annual cost per managed / unmanaged patient without complications. Reporting economic outcomes in model cost units helps provide standardized metric to evaluate relative intervention efficiency while preserving the underlying real-world capitation calibration of the Prolanis system.

Future costs and cumulative health outcomes were discounted at an annual rate of 3%, consistent with the Indonesian Health Technology Assessment guideline [26]. Undiscounted results using 0% and sensitivity results using 5% annual discounting were also generated to examine the robustness of the cost-effectiveness findings to the discount-rate assumption. Discounting was applied in the post-hoc economic evaluation of cumulative costs and cumulative outcomes. The epidemiological state trajectories and optimal-control paths were generated from the original deterministic optimal-control model and were not re-derived under a discounted mathematical objective function.

The primary effectiveness outcome for the Incremental Cost-Effectiveness Ratio (ICER) analysis was discounted cumulative managed patient-years, calculated by integrating the managed T2DM population over time after applying the annual discount factor. Endpoint managed population at each policy milestone was retained as a descriptive epidemiological outcome but was not used as the primary ICER denominator in the discounted base-case analysis. The discounted cumulative cost for each strategy was calculated as:

$$\:D{C}_{s}={\int\:}_{0}^{T}\left(\frac{\left({U}_{s}+{M}_{s}\right)+3.8{C}_{s}+{w}_{s}{u}_{s}}{{\left(1+r\right)}^{t}}\right)dt,$$

where $\:D{C}_{s}$ denotes the discounted cumulative cost for strategy (*\:s*) over time horizon (*\:T*). $\:{U}_{s},{M}_{s},{C}_{s}$ represent the unmanaged, managed and complication compartments respectively. $\:{w}_{s}$ is the intervention cost-weight, $\:{u}_{s}$ is the optimized intervention intensity, and *\:r* is the annual discount rate. In the base-case analysis, (*\:r=0.03*) corresponds to a 3% annual discount rate.

Discounted cumulative effectiveness was calculated as discounted managed patient-years:

$$\:D{E}_{s}={\int\:}_{0}^{T}\left(\frac{{M}_{s}}{{\left(1+r\right)}^{t}}\right)dt,$$

where $\:D{E}_{s}$ denotes the discounted cumulative managed patient-years generated by strategy (*\:s*) over time horizon (*\:T*). The ICER was then calculated as:

$$\:ICE{R}_{s,c}=\frac{D{C}_{s}-D{C}_{c}}{D{E}_{s}-D{E}_{c}},$$

where (*\:s*) represents the intervention strategy and (*\:c*) represents the comparator strategy.

Standard decision rules for ICER were applied to these total system costs. Any strategy resulting in higher systemic costs and fewer managed patients than a preceding strategy was classified as strictly dominated [28]. The non-dominated strategies were mapped onto a Cost-Effectiveness Plane (CEP) to visually determine their economic quadrants [29]. Because the base-case analysis demonstrated strict economic dominance for the optimal strategy under the specified model assumptions, parameter values, cost-weights, and 3% discounting, comparison against a willingness-to-pay ICER threshold was not required to establish cost-effectiveness.

### Time-horizon sensitivity analysis

Because the timing of capital outlays and averted complication penalties heavily influence health economic forecasting, a deterministic time-horizon sensitivity analysis was conducted [12, 17]. Upfront program implementation costs are incurred immediately, whereas the financial benefits of averting severe late-stage complications accrue over a delayed temporal trajectory.

The incremental discounted costs and discounted managed patient-years of all six strategies were re-evaluated at four discrete policy milestones ($\:T=5,\:10,\:15,\:25$). These specific time horizons were selected based on the mathematical dynamics of the disease:

- Year 5: Mathematical simulations indicate that the introduction of community interventions leads to a rapid increase in the managed T2DM population primarily within the first five years. Additionally, temporal sensitivity analyses demonstrate that intervention parameters exert their strongest mathematical influence on system dynamics during this early window [27].
- Year 10: A 10-year horizon is a universally accepted standard in clinical literature for projecting cardiovascular and cardiometabolic risks [30].
- Year 15: Evaluating the ICER at 15 years captures the intermediate trajectory between early implementation effects and the longer-term accumulation of irreversible late-stage complications.
- Year 25: This serves as the established maximum projection limit of the compartmental model, necessary to capture the total cumulative costs and observe the ultimate systemic outcome [31].

## Results

### Baseline 25-year cost-effectiveness analysis

To determine the most efficient resource allocation for community-based T2DM management, ICERs were calculated over a 25-year time horizon using a 3% annual discount rate for both cumulative costs and cumulative managed patient-years. Endpoint managed population was retained as a descriptive epidemiological outcome, whereas discounted cumulative managed patient-years were used as the primary effectiveness measure for the ICER analysis. Under the economic resource allocation framework, interventions with higher delivery cost-weights triggered budgetary penalties that severely restricted their feasible implementation intensity.

As summarized in Table 2, all active strategies generated higher discounted cumulative managed patient-years and lower discounted cumulative costs than baseline care under the specified model assumptions. However, lifestyle modification applied as a single intervention generated the largest absolute benefit, increasing the endpoint managed T2DM population by 0.50 million persons and producing an additional 12.74 million discounted managed patient-years compared with baseline. It also generated the largest discounted cost savings, reducing cumulative system cost by 19.22 model cost units over the 25-year horizon. Conversely, strategies that relied on traditional promotive group activities such as physical activity and health education generated smaller population effects and smaller cumulative cost savings.

**Table 2:** Discounted cumulative costs and health outcomes of community-based interventions at Year 25 under the 3% base-case discount rate

Strategy	Managed population, million	Incr. endpoint managed population vs. baseline, million	Disc. cumulative effect, million managed patient-years	Incr. discounted effect vs. baseline, million managed patient-years	Disc. cumulative cost, model cost units	Incr. discounted cost vs. baseline, model cost units
Baseline	0.475	Ref	32.857	Ref.	718.894	Ref
Physical activity only	0.479	0.004	33.14	0.283	718.577	-0.318
Health education only	0.483	0.008	33.434	0.577	718.246	-0.648
Lifestyle modification only	0.976	0.501	45.601	12.744	699.674	-19.22
Lifestyle modification + physical activity	0.507	0.032	35.19	2.333	716.256	-2.638
Lifestyle modification + health education	0.508	0.033	35.23	2.373	716.211	-2.683
Health education + physical activity	0.487	0.012	33.708	0.851	717.938	-0.957

Note: Costs are reported as discounted model cost units. If the original cost calibration is retained, these values may be interpreted using the manuscript’s IDR-trillion reporting scale. A negative ICER indicates that the strategy generated lower cumulative cost and higher cumulative effectiveness than baseline care

When the active strategies were compared directly with lifestyle modification only, all other strategies were strictly dominated because they produced fewer discounted managed patient-years while incurring higher cumulative costs (Table 3). This pairwise comparison supports the classification of lifestyle modification as the economically dominant strategy among the evaluated alternatives under the 3% base-case discount rate.

**Table 3:** Pairwise dominance analysis using lifestyle modification only as comparator at Year 25

Intervention	Comparator	Incremental cost, model cost units	Incremental effect, million managed patient-years	ICER	Decision
Baseline	Lifestyle modification only	19.22	-12.744	-1.508	Strictly dominated by lifestyle modification
Physical activity only	Lifestyle modification only	18.902	-12.46	-1.517	Strictly dominated by lifestyle modification
Health education only	Lifestyle modification only	18.572	-12.166	-1.526	Strictly dominated by lifestyle modification
Lifestyle modification + physical activity	Lifestyle modification only	16.582	-10.41	-1.593	Strictly dominated by lifestyle modification
Lifestyle modification + health education	Lifestyle modification only	16.537	-10.371	-1.595	Strictly dominated by lifestyle modification
Health education + physical activity	Lifestyle modification only	18.263	-11.893	-1.536	Strictly dominated by lifestyle modification

**Fig. 2:**
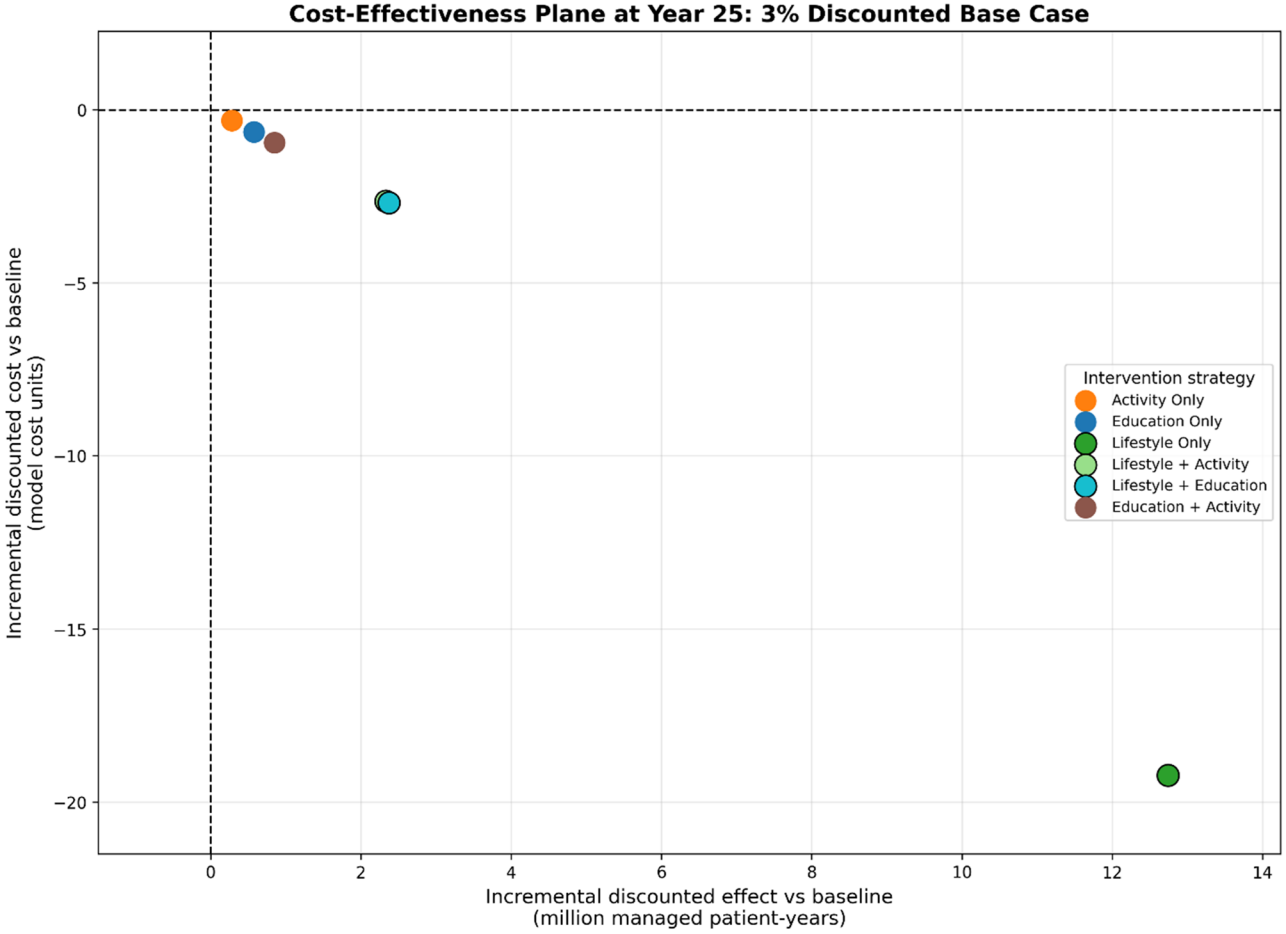
Cost-effectiveness plane at year 25

This economic dominance is visually shown in Fig. 2. The cost-effectiveness plane maps the incremental discounted costs and incremental discounted managed patient-years of the six active strategies against baseline care. Lifestyle modification only occupies the most favorable dominant position, combining the largest incremental effectiveness with the greatest cumulative cost savings. In contrast, the individual health education and physical activity strategies, as well as the combination strategies, remain closer to the origin, indicating smaller incremental gains and smaller cost savings under the same budget-constrained framework.

### Time-horizon sensitivity analysis

**Fig. 3:**
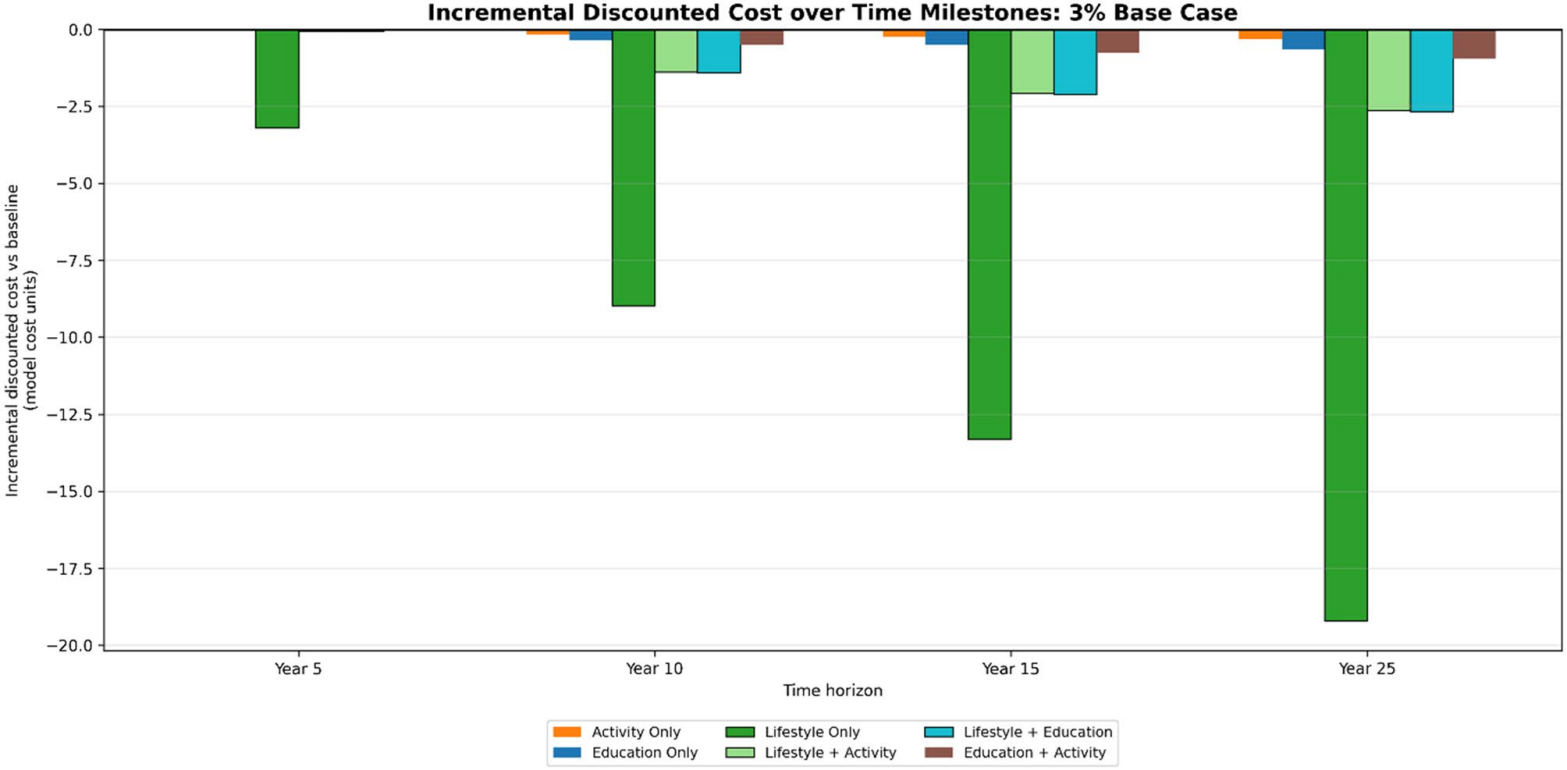
Incremental discounted cost over time milestones under the 3% Base-case discount rate

Because the timing of capital outlays and averted complication penalties heavily influence health economic forecasting, a time-horizon sensitivity analysis was conducted at discrete policy milestones. Figure 3 illustrates the incremental discounted cost variations relative to baseline care at Year 5, Year 10, Year 15, and Year 25.

- Year 5: The economic advantage of lifestyle modification was already evident within the first five years. Lifestyle modification only generated an additional 3.324 million discounted managed patient-years and reduced cumulative cost by 3.207 model cost units relative to baseline. This early effect reflects the modeled reduction in progression along the unmanaged pathway and the initial avoidance of complication-related cost penalties.
- Year 10 and 15: As the projection extended through the intermediate time horizons, the cumulative financial benefits of averted complications increased. Lifestyle modification only generated 6.681 additional discounted managed patient-years and 8.978 model cost units in savings by Year 10, increasing to 9.191 additional discounted managed patient-years and 13.311 model cost units in savings by Year 15. Combination strategies continued to show smaller cost savings and smaller health effects than lifestyle modification alone.
- Year 25: By the 25-year mark, lifestyle modification only produced the largest cumulative effect and cost savings, generating 12.744 additional discounted managed patient-years and 19.22 model cost units in savings compared with baseline. The temporal analysis therefore indicates that, under the model assumptions and cost-weight structure, prioritizing monitoring-supported lifestyle modification provides the most favorable long-term economic trajectory among the evaluated Prolanis-derived strategies.

Discount-rate sensitivity analysis supported the robustness of this finding (Table 4). Under the undiscounted scenario 0%, lifestyle modification only generated 17.783 million additional managed patient-years and 27.610 model cost units in savings relative to baseline. Under the 5% discount-rate scenario, it remained dominant, generating 10.481 million additional discounted managed patient-years and 15.476 model cost units in savings. These results indicate that the dominance of lifestyle modification was not dependent on the 3% base-case discount-rate assumption.

**Table 4:** Discount-rate sensitivity analysis for lifestyle modification only versus baseline at Year 25

Discount Rate	Incremental cost vs. baseline, model cost units	Incremental effect vs. baseline, million managed patient-years	ICER	Decision
Undiscounted 0%	-27.61	17.783	-1.553	Dominant vs. baseline
Baseline 3%	-19.22	12.744	-1.508	Dominant vs. baseline
Sensitivity 5%	-15.476	10.481	-1.477	Dominant vs. baseline

## Discussion

### The economic dominance of lifestyle modification

The cost-effectiveness plane confirms that lifestyle modification was the most economically favorable strategy under the 3% discounted base-case analysis, producing the largest gain in discounted managed patient-years while generating the greatest cumulative cost savings. This finding should be interpreted conditionally on the model structure, parameter values, cost-weights, and payer-perspective assumptions used in the analysis. Compared with baseline care, lifestyle modification generated 12.744 additional discounted managed patient-years and reduced cumulative system cost by 19.22 model cost units over the 25-year horizon. Pairwise dominance analysis further showed that all other active strategies were less effective and more costly than lifestyle modification alone, supporting its classification as the dominant strategy among the evaluated alternatives.

Beyond the mathematical representation, this dominance is supported by plausible public-health mechanisms. Monitoring-supported lifestyle modification combines routine assessment of clinical markers and health-status monitoring within primary-care workflows. This mechanism can support earlier detection of poor glycemic control or emerging complications, facilitate timely treatment adjustment, reinforce self-management behavior, and delay progression toward late-stage complications. By shifting patients from the unmanaged compartment to the managed compartment, this strategy reduces exposure to the higher unmanaged-to-complication transition pathway and increases the time patients spend in the lower-risk managed state.

On the other hand, traditional promotive group activities, including health education and physical activity, cluster closer to the origin (0,0) representing the baseline care comparator on the cost-effectiveness plane, indicating smaller incremental gains and smaller cost savings under the same budget-constrained framework. This does not imply that health education and physical activity lack clinical or public-health value. Rather, under the specified resource allocation model, their higher implementation cost-weights restrict feasible intensity and reduce their population-level economic efficiency when they are implemented as standalone or combined strategies.

A notable finding of this study is the suboptimal performance of combination strategies under strict budgetary constraints. Figure 2 shows that any combination involving health education or physical activity performs worse than lifestyle modification alone. This finding is explained by the economic resource allocation framework. According to Khoe et al. (2020), group-based interventions like health education and physical activity require substantially higher delivery cost-weights than routine monitoring [8]. When these expensive pillars are bundled into a combination strategy, their high fiscal weight triggers mathematical cost penalties within the objective function to maintain realistic resource allocation boundaries. Consequently, under the model assumptions, bundling higher-cost activities may dilute the available primary-care budget and produce smaller population-level gains than delivering low-cost monitoring-supported lifestyle modification at higher feasible intensity.

The Time-Horizon Sensitivity Analysis further reinforces the viability of the lifestyle modification intervention by mapping exactly when the intervention offsets its implementation costs. In this model, lifestyle modification generated net savings by Year 5 and maintained economic dominance through Years 10, 15, and 25. Discount-rate sensitivity analysis further supported this conclusion: lifestyle modification remained dominant under both the undiscounted scenario and the 5% discount-rate scenario, indicating that the finding was not driven solely by the 3% base-case discount-rate assumption. Because the direct medical cost of treating a T2DM patient with complications is approximately 3.8 times higher than treating one without complications, the early and sustained implementation of lifestyle monitoring continuously accumulates systemic cost savings.

### Comparison with existing evidence and policy implications

In the national context, Indonesia’s primary care preventive budget is increasingly constrained by the rising costs of complications [8]. Recent evaluations of the Prolanis program at the Sibolga Branch Office reveal that programs which bundle group health education and physical activity consistently account for the vast majority of the Prolanis budget, consuming up to 66.4% of total expenditure annually from 2020 to 2022 [32]. 

While this represents a localized success case for group activities, broader evidence suggests this model is not easily representative nationally [33]. Across many regions, broader evaluations and scoping studies summarize that implementation is frequently hindered by geographic barriers, workforce shortages, socio-economic challenges and facility-level constraints [8, 33, 34]. 

If the preventive budget share remains limited and infrastructure-constrained in most primary care sites, our finding suggests that the most economically efficient policy lever is low-cost, monitoring-supported lifestyle management. This finding aligns with recent health economics modeling studies of community-based T2DM prevention in Indonesia, which similarly emphasize that continuous monitoring and early risk detection are highly cost-effective strategies for averting long-term complications [35]. Therefore, to improve long-term financial sustainability, policymakers should consider gradually rebalancing Prolanis capitation allocations toward continuous monitoring as the core scalable component.

While lifestyle modification is the most cost-effective on a broad population scale, group-based education and physical activity may remain clinically important for specific demographic or geographic subgroups, particularly elderly, rural, or low-literacy patients who may benefit from structured contact, peer support, and supervised community engagement. The policy implication is therefore one of strategic resource reallocation rather than replacement. Lower-cost monitoring-supported lifestyle management may be prioritized as the core scalable component, while group activities may be targeted to populations in whom their incremental value is likely to justify the additional implementation cost.

### Contribution to health-economic literature

As stated in the introduction, a major gap in existing health-economic evidence is the limited availability of long-term, policy-relevant economic forecasting for community-based diabetes programs in LMIC settings. This study contributes to that gap by extending a previously developed non-reversible deterministic T2DM compartmental model into a health-economic framework that incorporates direct medical costs, complication-cost penalties, discounting, ICER-based decision rules, and time-horizon sensitivity analysis. The explicit irreversibility assumption is important because it prevents the model from overstating recovery from advanced complication states and provides a conservative structure for evaluating long-term complication costs. By integrating Indonesian epidemiological evidence with payer-perspective cost inputs, the model provides a mathematical framework for evaluating how Prolanis resources could be allocated under realistic fiscal constraints.

### Study limitations

While this study provides mathematical forecasting for T2DM management, several limitations must be acknowledged. First, the economic evaluation was conducted strictly from BPJS Kesehatan perspective, capturing only direct medical and program delivery costs. As highlighted by recent systematic reviews of T2DM economic burdens in Indonesia, economic evaluations frequently omit indirect costs as well as intangible costs [25]. Consequently, the total economic savings generated by averting complications through lifestyle modification intervention are likely underestimated in this model.

Second, the projected decline in the absolute managed population at Year 25 across all states reflects the non-reversible structure of the compartmental framework, where individuals progressively exit the managed state into advanced complication states or natural mortality. This deterministic model strictly relies on the assumption of irreversible disease progression to avoid overestimating the clinical benefits of the primary care interventions. While this conservative approach provides a robust baseline for long-term policy forecasting, it simplifies individual-level clinical heterogeneity and dynamic behavioral feedback mechanisms. Individual-level adherence was not explicitly modeled as a time-varying behavioral process. Instead, the transition parameters and intervention modifiers represent population-level effects calibrated from secondary data, which may not capture long-term behavioral fatigue or individual implementation drop-off.

Third, the unit cost parameters used for the interventions were derived from aggregated national and regional data. This may not fully capture the vast geographic and infrastructural barriers across Indonesia’s Prolanis program [33]. Because Indonesia has substantial interregional variation in primary-care capacity, transportation access, workforce availability, and patient engagement, the relative cost-effectiveness of each Prolanis component may differ across urban, rural, and remote settings.

Finally, while our resource allocation framework mathematically shows that combining expensive interventions dilutes budget intensity, future work should explore whether emerging digital health technologies could lower the unit costs of these interventions, potentially making combination strategies more financially viable. Future work should also evaluate disaggregated complication states, probabilistic parameter uncertainty, equity impacts, and broader societal costs, including productivity losses and caregiver burden.

## Conclusions

This study provides a payer-perspective health-economic extension of a non-reversible deterministic T2DM compartmental model to evaluate Prolanis-derived community-based interventions in Indonesia. Under the specified model assumptions, delivery cost-weights, and 3% discounted base-case analysis, monitoring-supported lifestyle modification was the dominant strategy, producing the greatest gain in discounted managed patient-years and the largest cumulative cost savings over 25 years. This finding remained stable under 0% and 5% discount-rate sensitivity analyses.

The results support a strategic resource reallocation of Prolanis capitation resources toward continuous, low-cost, monitoring-supported lifestyle management as the scalable core intervention across primary care facilities. However, health education and physical activity should not be interpreted as ineffective or unnecessary. Rather, group-based interventions should be strategically targeted toward specific demographic populations, including elderly, rural, or low-literacy patients, where their clinical value justifies the higher implementation cost-weight.

These findings should be interpreted considering model limitations, including the absence of explicit individual-level adherence, long-term behavioral fatigue, intervention fidelity, probabilistic uncertainty, indirect costs, and disaggregated complication states. Future work should incorporate longitudinal implementation data, regional cost variation, adherence scenarios, and broader societal costs to refine resource-allocation decisions within Indonesia’s chronic disease management program.

## Data Availability

The data that support the findings of this study are subject to a non-disclosure agreement and therefore cannot be shared publicly. Indonesian Health Survey 2023’s data may be accessed upon reasonable request and completion of the required non-disclosure agreement through the Ministry of Health of the Republic of Indonesia’s official platform at https://www.badankebijakan.kemkes.go.id/en/data-mikro-ski.
